# Rapid Inkjet-Printed Miniaturized Interdigitated Electrodes for Electrochemical Sensing of Nitrite and Taste Stimuli

**DOI:** 10.3390/mi12091037

**Published:** 2021-08-28

**Authors:** Sohan Dudala, Sangam Srikanth, Satish Kumar Dubey, Arshad Javed, Sanket Goel

**Affiliations:** 1 MEMS, Microfluidics and Nanoelectronics Lab, Department of Electrical and Electronics Engineering, Birla Institute of Technology and Science Pilani, Hyderabad Campus, Hyderabad 500078, India; h20171060103@hyderabad.bits-pilani.ac.in (S.D.); p20180451@hyderabad.bits-pilani.ac.in (S.S.); satishdubey@hyderabad.bits-pilani.ac.in (S.K.D.); arshad@hyderabad.bits-pilani.ac.in (A.J.); 2 Department of Mechanical Engineering, Birla Institute of Technology and Science Pilani, Hyderabad Campus, Hyderabad 500078, India

**Keywords:** inkjet printing, microfabrication, nitrite sensing, taste sensing

## Abstract

This paper reports on single step and rapid fabrication of interdigitated electrodes (IDEs) using an inkjet printing-based approach. A commercial inkjet-printed circuit board (PCB) printer was used to fabricate the IDEs on a glass substrate. The inkjet printer was optimized for printing IDEs on a glass substrate using a carbon ink with a specified viscosity. Electrochemical impedance spectroscopy in the frequency range of 1 Hz to 1 MHz was employed for chemical sensing applications using an electrochemical workstation. The IDE sensors demonstrated good nitrite quantification abilities, detecting a low concentration of 1 ppm. Taste simulating chemicals were used to experimentally analyze the ability of the developed sensor to detect and quantify tastes as perceived by humans. The performance of the inkjet-printed IDE sensor was compared with that of the IDEs fabricated using maskless direct laser writing (DLW)-based photolithography. The DLW–photolithography-based fabrication approach produces IDE sensors with excellent geometric tolerances and better sensing performance. However, inkjet printing provides IDE sensors at a fraction of the cost and time. The inkjet printing-based IDE sensor, fabricated in under 2 min and costing less than USD 0.3, can be adapted as a suitable IDE sensor with rapid and scalable fabrication process capabilities.

## 1. Introduction

Interdigitated electrodes (IDEs) have gained significant popularity since their introduction in the late 1990s. Interdigitated electrodes, also referred to as Interdigitated arrays (IDAs), are high aspect ratio electrodes closely spaced together to form an array of sensing regions which significantly increase the effective area available for electrochemical sensing. IDEs find extensive use and application in electronic sensor and actuator applications along with biological and chemical sensing applications. Biological sensing using IDEs includes DNA sensing, viral load detection, label-free detection systems, etc. [1,2,3]. Volatile organic compound sensing, gas sensing, and sensing of harmful chemical components are a few of the many applications of IDEs in chemical sensing [4,5,6,7]. Due to the growing trend towards miniaturized systems, IDEs even find application in energy storage and lab-on-a-chip devices [8,9].

Interdigitated electrodes are often employed for the electrochemical impedance spectroscopy (EIS) technique for quantitative sensing [10]. EIS is used to analyze the impedance behavior of the system at applied potential with varying frequencies and finds application in various fields including chemical analysis [11,12], study of fuel cells and batteries [13,14,15], corrosion analysis [16], etc. Total impedance (Z), represented as a combination of a real part and an imaginary part, is an important parameter for analyzing electrode–electrolyte interactions in aqueous systems. The real part of the impedance represents the resistance, while the imaginary part represents the reactance. EIS, with the aid of IDEs, offers increased sensitivity due to the increased sensing surface area. Most of such IDEs are manufactured using complex fabrication processes, often involving lengthy and skill-intensive photolithography processes, mostly in cleanroom environments [17,18]. At times, extensive surface modification is required to improve the sensing capabilities of the fabricated IDEs [19]. However, with emerging materials, such as polymer films, highly conductive inks, etc., coupled with new and inexpensive fabrication techniques, fabrication of IDEs, sensors, and electronics is being simplified [20,21,22,23,24,25,26,27,28].

IDEs are extensively used for various sensing applications, including environmental monitoring such as soil health monitoring, water quality assessment, air quality measurement, etc. One of the essential environmental monitoring applications is water quality assessment where various methods, such as colorimetric, fluorometric, electrochemical, etc., have been reported [29,30,31,32]. Electrochemical assessment of water quality using IDEs can overcome issues associated with optical detection systems which require optically clear samples. IDEs have been employed for the detection of various components and contaminants in water, including salinity and ions [24,33]. Nitrite is also a contaminant found in water obtained from unchecked sources which, when consumed in excess via contaminated water sources, can have adverse effects on humans [34,35]. In this context, nitrite detection has been demonstrated as a possible application for fabricated IDEs in this work. Another application where IDEs are employed is taste sensing. Human taste buds are capable of sensing and differentiating between five fundamental tastes—namely, salty, sour, bitter, sweet, and umami. For experimental purposes, herein, five chemicals have been identified which can replicate these fundamental tastes; they are sodium chloride for salty, L-tryptophan for bitter, guanosine monophosphate (GMP) for umami, citric acid for salty, and sucrose for sweet [36]. In a recent work, a flexible IDE sensor was developed using laser-induced graphene [36]. However, the reported fabrications are a two-step process involving the development of laser-induced graphene followed by the transfer of developed graphene onto a Kapton tape. The process seems to be effective, but the process of transfer might result in a partial transfer of film, making the sensor less reliable. A single step process for fabrication could improve the repeatability in sensor fabrication.

In this work, a single step, rapid miniaturized IDE sensor fabrication process, using an inkjet printing approach, is reported. A commercial inkjet PCB printer has been employed for fabrication of the IDEs on a glass substrate. Carbon ink, adjusted for viscosity to enable use with the printer, was used and characterized post-printing. The inkjet-printed IDE’s performance was compared with that of an IDE fabricated using a maskless direct laser writing (DLW)-based photolithography. Two types of chemical analysis studies were conducted to establish the functionality of the IDE sensors developed using inkjet printing. The following sections will elaborate on the design, modelling, experimental methodology, and the observations made using the fabricated interdigitated electrode sensors.

## 2. Materials and Methods

### 2.1. Materials and Equipment

Chemicals conforming to analytical grade were used. Guanosine-5-monophosphate disodium salt (GMP), citric acid anhydrous, l-tryptophan, sucrose, and sodium chloride were procured from Sisco Research Laboratories Pvt. Ltd. (Mumbai, India). Sodium nitrite and acetone were obtained from Sigma-Aldrich (St. Louis, MO, USA). Sodium carbonate was procured from TCI Chemicals Pvt. Ltd. (Tokyo, Japan). Ethanol was obtained from Changshu Hongsheng Fine Chemicals Co. Ltd. (China). All solutions were prepared in Milli-Q water (18.2 MΩ.cm) at room temperature (≈25 °C).

Borosil glass slides measuring 75 mm × 25 mm × 1 mm were used as substrates for inkjet printing. The conductive carbon ink (viscosity: 25,000 cps) was obtained from Engineered Materials Systems, Inc. (EMS) (Delaware, OH, USA). Negative dry film photoresist of 1.5 mil, i.e., 40 µm thickness (RistonPM 240, Dupont, Wilmington, DE, USA), was used for photolithography. The V-One inkjet-printer (Voltera, Kitchener, Canada) and direct laser writing system (HO-LWS-PUV, Holmarc, Kochi, India) were used for fabrication.

### 2.2. Design and Modelling of Interdigitated Electrodes (IDEs)

Prior notable works have been carried out to model and optimize interdigitated electrodes for impedance-based sensing [37,38,39]. The design parameters, limiting factors for selection of those parameters, and their optimized values have been thoroughly discussed in this work. For representing systems incorporating the use of IDEs for analysis of analytes in a solution, the equivalent model proposed by Hong et al. [39] is widely employed. This model was referred to for selecting and optimizing design parameters for IDEs for the required immersed sensing application. The primary consideration for selecting the overall dimensions of the electrode was the dimensions of the substrate (microscope slide) for inkjet printing. The length of each finger was selected based on the width of such glass substrate. The lower limiting value for selecting the width of the IDE was limited by the nozzle thickness of Voltera V-One. The width of each electrode was selected to be 310 µm which was found to be the most repeatable single-width capable of being printed using the inkjet printing.

**Figure 1:**
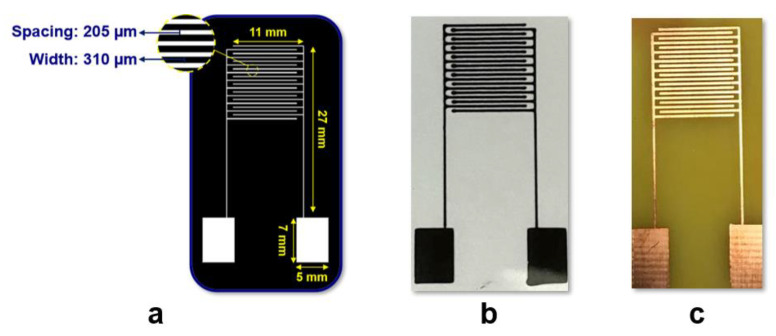
(**a**) Optimized Design (**b**) Carbon ink-based IDE printed on glass slide (**c**) Photolithography-based DLW IDE on a copper-cladded FR4 board.

The sensitivity of IDE sensors can be evaluated using the contact surface between the sample solution and electrodes. The metallization ratio (a), which is the ratio of space between the successive fingers and the width of the electrodes, plays an important role in determining the sensitivity for IDE-based sensing applications. The analytically optimized value of the metallization ratio, i.e., a = 0.66, was used [38]. Based on the selected width of the electrodes, a gap of 205 µm between each finger was derived. Another parameter required to be selected was the number of fingers which plays an important role in deciding the cell constant of the cell. A lower cell constant is preferred, which can be achieved by increasing the number of fingers (n). It was analytically observed that beyond 20 fingers, no appreciable change in cell constant was observed [38]. Further, as 20 fingers could be easily accommodated on the glass substrate, *n* = 20 was selected for designing and fabricating the IDEs. Post the selection of geometric parameters, the interdigitated electrode arrays were designed in LayoutEditor. Finger spacing and electrode widths of 205 µm and 310 µm were used, respectively, with a total of 20 fingers for the CAD model. Contact pads of 5 mm × 7 mm (Figure 1a) were also incorporated in the design to enable clipping of alligator clips during EIS analysis.

### 2.3. Fabrication of Inkjet Printing-Based (IDE) Sensor

**Figure 2:**
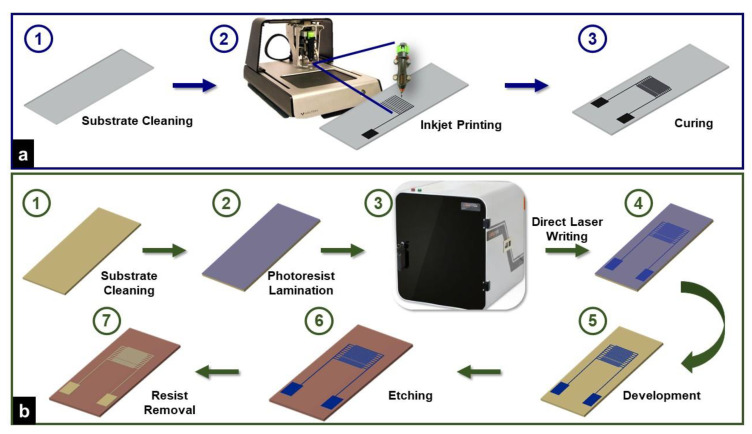
Overall step-by step fabrication scheme for (**a**) Carbon ink-based IDE printed on glass slide (**b**) Photolithography-based DLW IDE on a copper-cladded FR4 PCB board.

Figure 2a describes the overall fabrication scheme of the inkjet printing-based IDE sensor. The software bundled with Voltera V-One recognizes designs as vector image files in Gerber format. Thus, the design created in LayoutEditor was converted to Gerber format using DipTrace. Voltera V-One (Figure 2(a2)) uses a nozzle-cartridge-based printing approach. The cartridge can be filled with custom viscous pastes. Prior to use, the supplied cartridges were thoroughly washed with acetone. The cartridge was filled with the viscous carbon ink after the ink’s viscosity was adjusted using acetone and the cartridge was primed to ensure adequate and continuous operation. The layout verification, height calibration of bed, and substrate was conducted using the supplied probe. The primed cartridge was replaced with the probe. After multiple trials, optimized parameters for the best printing conditions were used. Nozzle height, which is the space between the substrate and the ink nozzle, was optimized at 150 µm, and the space between the probe and the substrates was set as 0.13 mm. Dispensing amount, which is a machine parameter to control the amount of ink being extruded out of the nozzle, was set to 50. A glass substrate, thoroughly cleaned with ethanol, was used. Post printing of IDEs on the glass substrate (Figure 1b), heat treatment was carried out at 100 °C for 20 min.

### 2.4. Fabrication of DLW-Photolithography-Based (IDE) Sensor

The complete fabrication process for the DLW-photolithography-based IDE sensor is schematically represented in Figure 2b. A customized direct laser writing (DLW) system (Figure 2(b3)) was used for maskless photolithography fabrication. The design created using LayoutEditor was imported into the software of the DLW system. The DLW system uses a 405 nm GaN laser diode with a maximum intensity of 65,000 W/m^2^. An FR-4 copper-cladded board was coated with a negative dry film photoresist (DFR) using a hot roll laminator (A3 Mega Drive Laminator, Cambridge, UK). Optimized laser writing parameters, such as speed, focal distance, etc., selected from our previous work performed on DLW system optimization [40,41] were utilized to obtain a width of 310 µm. Post laser writing, the resist development was conducted using 0.85% sodium carbonate. The etching of unprotected copper was completed using ferric chloride. The final step involved resist removal, a rinse with Milli-Q water and acetone, and air drying. Figure 1c shows the IDE fabricated using DLW-photolithography.

### 2.5. Experimental Setup

First, various stock solutions of different analytes were prepared. A 50 ppm stock solution of sodium nitrite was prepared. Sodium nitrite stock solution was serially diluted to obtain solutions of concentrations—1, 10, 20, and 50 ppm. For taste simulating chemicals, stock solutions of 500 ppm were each prepared for guanosine-5-monophosphate disodium salt (GMP), L-tryptophan, sucrose, citric acid, and sodium chloride. Each of these chemicals were further diluted to concentrations of 1, 5, 10, 50, 100, 150, and 250 ppm for concentration analysis. All the prepared standard solutions were used for EIS analysis using both types of fabricated sensors.

**Figure 3:**
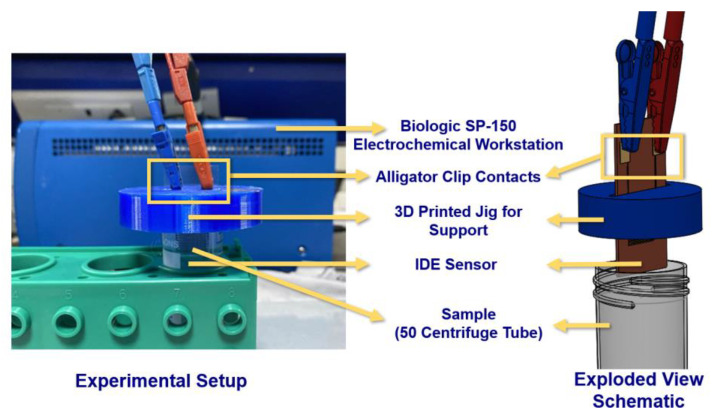
Experimental setup and schematic showing the enhanced view of the experimental setup for EIS analysis using potentiostat. A two-electrode configuration was employed with a frequency ranging from 1 Hz to 1 MHz.

The electrochemical workstation (SP-150 from Biologic, France) was used to perform electrochemical impedance spectroscopy (EIS) in two electrode configurations. To avoid temperature-induced effects, it was ensured that all experiments were conducted at similar room temperature (~24 °C). The frequency and voltage ranges were set as 1 Hz–1 MHz and ±10 V, respectively. To avoid any unnecessary interference, separate unused IDE sensors were used for each type of chemical. For different concentrations of the same chemical, a water–ethanol cleaning protocol was employed. The IDE was rinsed with Milli-Q water, air dried for 20 min at 70 °C, rinsed with ethanol, and air dried at room temperature for 5 min before each use. Electrochemical workstation probes were connected to IDEs using alligator clips to ensure tight and continuous contact with the contact pads. A 3D printed jig was used to hold the IDE in place and prevent the electrochemical workstation probes from coming in direct contact with the analyte solution (Figure 3).

## 3. Results and Discussion

### 3.1. Characterization of Inkjet Printing-Based (IDE) Sensor

**Figure 4:**
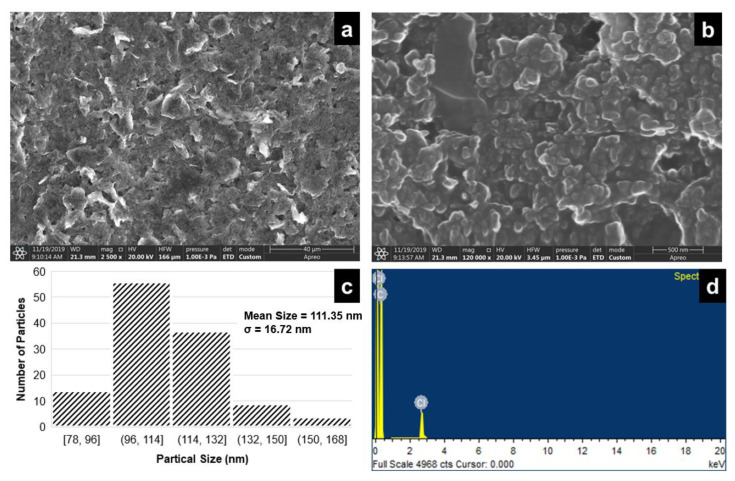
SEM image of inkjet-printed carbon ink at a magnification scale corresponding to (**a**) 40 µm, (**b**) 500 nm, (**c**) particle size distribution plot (mean particle size = 111.35 nm and standard deviation, σ = 16.72 nm) and (**d**) EDX analysis.

Scanning electrode microscopy (SEM) (Apreo scanning electron microscope, Thermo Fisher Scientifc, Waltham, MA, USA) and energy-dispersive X-ray (EDX) spectroscopy were performed to characterize the carbon inkjet-printed IDE (Figure 4). SEM images at different magnifications showed good porosity of the carbon paste. Further, the EDX analysis showed a good weight percentage of carbon (95.94%). Figure 4c represents the particle size distribution. The mean size of the particles was found to be 111.35 nm and the standard deviation for the particle size was found to be 16.72 nm.

**Figure 5:**
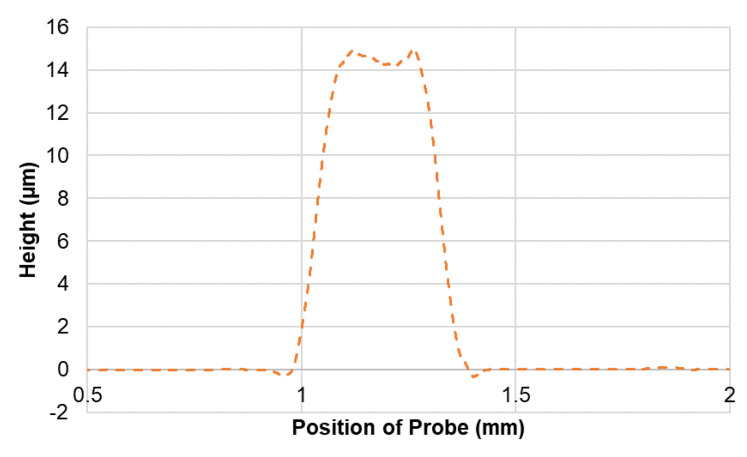
Profile obtained for thickness of single finger of inkjet printing-based (IDE) sensor measured using a stylus profiler.

A stylus profiler with a 2 µm tip (DektakXT, Bruker, MA, US), was used to find the thickness of the inkjet-printed layers. Figure 5 represents the profile obtained for the thickness of a single finger of the inkjet printing-based (IDE) sensor. The thickness was measured at four different locations and the average height was found to be 14.51 µm. The conductivity and resistivity measurements of the inkjet-printed electrode layer were conducted using a four-point probe system (Ossila, Sheffield, UK). The resistivity and conductivity were found to be (3.2030 ± 0.0094) x 10^−4^ Ω.m and 3122.079 ± 9.189 S/m, respectively (*n* = 50).

### 3.2. Chemical Sensing

Two types of chemical analysis were carried out in this work—nitrite quantification in aqueous solutions and detection of taste simulating chemicals. An EIS study for the prepared standard solutions was carried out. Frequencies from 1 Hz to 1 MHz were used for the EIS analysis. However, depending on the type of analysis, only certain relevant portions of the whole analysis were considered.

**Figure 6:**
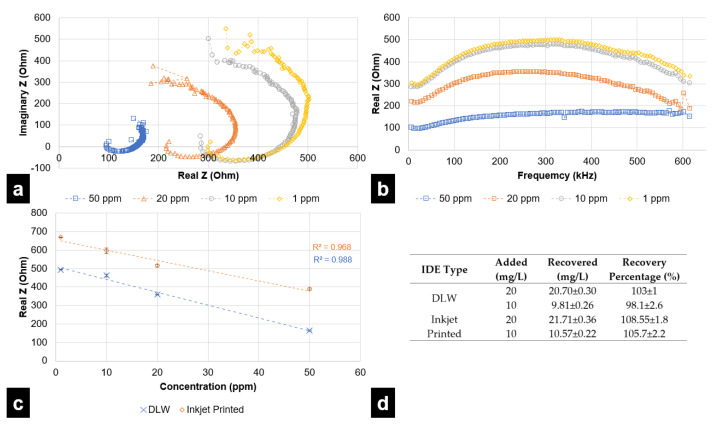
(**a**) Nyquist plot for nitrite sensing using the DLW-photolithography-based IDE sensor for different concentrations (**b**) real part of impedance vs. frequency plot for DLW-photolithography-based IDE sensor showing the nature of resistive impedance maximizing at 265 kHz (**c**) results derived from EIS analysis for nitrite concentration evaluation using inkjet printing-based IDE Sensor and DLW-photolithography-based IDE sensor in terms of resistive impedance vs. concentration (**d**) results for the analysis of tap water for detection via recovery of nitrite at different concentrations.

Standard solutions of sodium nitrite of different concentrations (1, 10, 20, and 50 ppm) were used for the EIS analysis. Three trials each (*n* = 3) were carried out for each type of sensor, i.e., inkjet printing-based IDE sensor and DLW-photolithography-based IDE sensor at each standard solution concentration. From the EIS analysis, regions corresponding to maximum values of real and imaginary parts of impedance where the sensor displays a sensitive nature were identified. For the inkjet printing-based IDE sensor, such frequencies were identified to be 97 kHz and 331 kHz, respectively, for real and imaginary parts. In the case of the DLW-photolithography-based IDE sensor, these frequencies were identified as 265 kHz and 615 kHz for the real and imaginary parts of impedance, respectively. Figure 6a represents the Nyquist plots for nitrite sensing in the range of 1–50 ppm for the DLW-photolithography-based IDE sensor. The maximum value for the imaginary part of impedance was observed at the end of the scan and was still increasing in the end. Therefore, the real part of impedance was selected for the relative comparison of sensors. Figure 6b clearly shows the varying nature of the real part of impedance versus the frequency at different concentrations. From Figure 6b, it can be clearly observed that the value of resistive impedance reaches its limiting value for each concentration at 265 kHz. To establish a comparison between DLW-photolithography and inkjet printing-based IDE sensors, the real part of impedance at their respective identified frequencies for maximum value were used. Based on the obtained fitted linear curve, the limit of detection [30] and other relevant parameters were estimated for both types of sensors and reported in Table 1. Table 1 also provides a comparison of the two types of sensors’ merits. The DLW-photolithography-based IDE sensor demonstrates better LOQ and LOD but the inkjet printing-based IDE sensor offers a rapid and low-cost fabrication. Both sensors can effectively quantify nitrite in water with good repeatability up to 1 ppm, which is three times lower than the WHO recommended limiting value of 3 ppm for potable water [30,34]. To analyze selectivity of both the sensors for nitrite detection and quantification, tap water was spiked with known concentrations of nitrite solution and samples were subjected to testing using the developed IDEs. The results tabulated in Figure 6d indicate good recovery percentages, thus establishing that the developed sensors can be used for real-life applications with selectivity towards nitrite.

For each simulated taste chemical, data is represented through two plots—(a) real Z (i.e., resistance) vs. frequency and (b) imaginary Z (reactance) vs. frequency. Even though the analysis was performed up to 1 MHz, results attained saturation after a specified frequency. Therefore, results up to 69 kHz have been considered for the DLW-photolithography-based IDE sensor. For the inkjet printing-based IDE sensor, frequencies up to 62 kHz have been considered. During experimental trials, it was observed that concentrations lower than 100 ppm were not effectively quantified and differentiated by the fabricated inkjet printing-based sensor.

**Figure 7:**
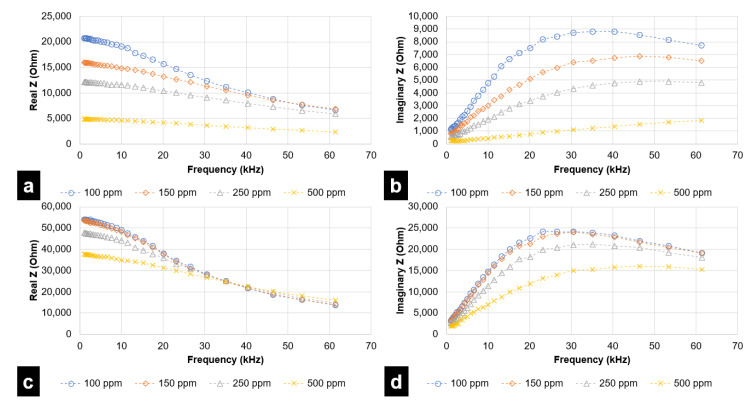
EIS analysis for Citric Acid (sour) and L-tryptophan (bitter), in the range of 1 kHz to 62 kHz, for inkjet printing-based IDE sensor in terms of (**a**) Resistive Impedance vs. Frequency (Citric Acid) (**b**) Reactive Impedance vs. Frequency (Citric Acid) (**c**) Resistive Impedance vs. Frequency (L-tryptophan) (**d**) Reactive Impedance vs. Frequency (L-tryptophan).

**Figure 8:**
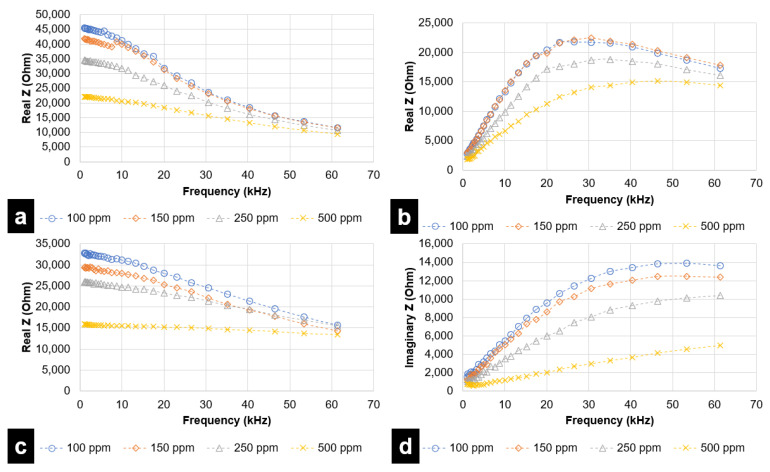
EIS analysis for GMP (umami) and sucrose (sweet), in the range of 1 kHz to 62 kHz, for inkjet printing-based IDE sensor in terms of (**a**) Resistive Impedance vs. Frequency (GMP) (**b**) Reactive Impedance vs. Frequency (GMP) (**c**) Resistive Impedance vs. Frequency (sucrose) (**d**) Reactive Impedance vs. Frequency (sucrose).

**Figure 9:**
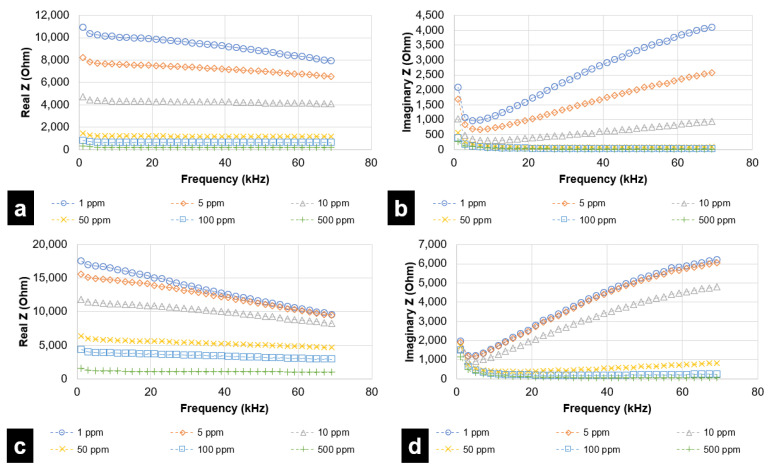
EIS analysis for Citric Acid (sour) and L-tryptophan (bitter), in the range of 1 kHz to 69 kHz, for DLW-photolithography-based IDE sensor in terms of (**a**) Resistive Impedance vs. Frequency (Citric Acid) (**b**) Reactive Impedance vs. Frequency (Citric Acid) (**c**) Resistive Impedance vs. Frequency (L-tryptophan) (**d**) Reactive Impedance vs. Frequency (L-tryptophan).

**Figure 10:**
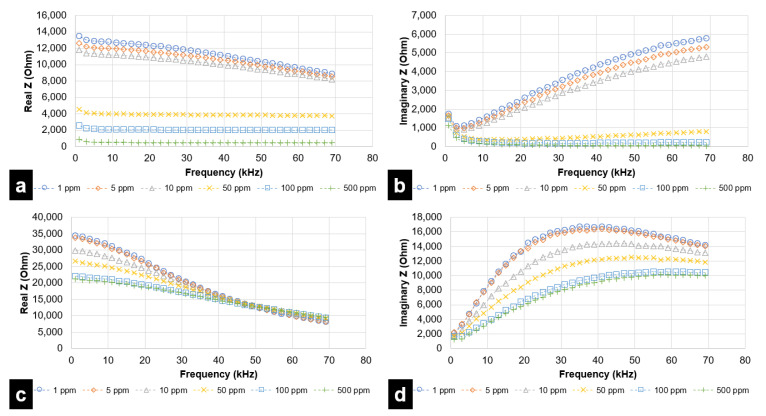
EIS analysis for GMP (umami) and sucrose (sweet), in the range of 1 kHz to 69 kHz, for DLW-photolithography-based IDE sensor in terms of (**a**) Resistive Impedance vs. Frequency (GMP) (**b**) Reactive Impedance vs. Frequency (GMP) (**c**) Resistive Impedance vs. Frequency (sucrose) (**d**) Reactive Impedance vs. Frequency (sucrose).

Figure 7 and Figure 8 represent the response of the inkjet printing-based IDE sensor with various taste simulating chemicals. Figure 9 and Figure 10 depict results for sensing using the DLW-photolithography-based IDE sensor. As expected, based on the model described by Hong et al., the resistivity (real Z) increases at higher frequencies. The change in impedance values at varying frequencies is evident from the plots. The solution resistance and double-layer capacitance play a vital role in describing EIS behaviors. The solution resistance results lead to an increase in resistance at higher frequencies. Reactance (imaginary Z) behavior is dependent on double-layer capacitance, amongst other parameters. As evident from Figure 7b, the imaginary part of impedance (reactance) increases with increases in frequency to around 35 kHz, which can be attributed to the charge separation at the electrode–electrolyte interface. This is followed by a steady decrease in reactance due to double-layer shortening. The impedance behavior for both L-tryptophan and GMP was found to be similar, which could be attributed to the analogous chemical structures comprising amino and carboxyl groups in both of them. At higher frequencies, polarization of oxygen atoms takes place in both carboxyl groups and phosphate groups (which is present in GMP). This leads to an overall increase in charge carriers, causing a change in the reactance values at higher frequencies. Solution resistance governs the resistive behavior for both GMP and L-tryptophan. Increased ion-solvent interactions at higher frequencies in the presence of sucrose is responsible for the resistance behavior. As in previous instances, double-layer capacitance largely governs the reactance behavior of sucrose.

A DLW-photolithography-based IDE sensor was primarily used to provide a performance reference for the inkjet printing-based IDE sensor. Figure 9 and Figure 10 represent the EIS analysis results for the DLW-photolithography-based IDE sensor. The trends observed for the respective chemicals were found to be very similar to that in the case of the inkjet printing-based IDE sensor. However, it is clearly evident that DLW-photolithography-based IDE sensors can effectively sense lower concentrations, as low as 1 ppm. Unlike in the case of citric acid (Figure 9a,b), for L-tryptophan (Figure 9c,d), GMP (Figure 10a,b), and sucrose (Figure 10c,d), the data points for resistance and reactance are closely spaced for lower concentrations at 1, 5 and 10 ppm. Though sensing at lower concentrations has been demonstrated, the resolving performance of the DLW-photolithography-based IDE sensor at lower concentrations needs improvement.

An important shortcoming in the application of both types of IDE sensors for taste sensing applications was the inability to sense for the presence of sodium chloride. Due to the electrolytic behavior of both sodium chloride and citric acid, it was expected that a similar trend would be observed with a change in absolute resistive and reactance values owing to the highly conductive nature of sodium chloride. However, with the set EIS analysis parameters, no reportable results were observed for sodium chloride. This inability to quantify sodium nitrite could be attributed to the highly polarizing nature of the analyte when compared with other analytes.

## 4. Conclusions

Herein, a simple, rapid, and single step interdigitated electrode fabrication process has been discussed and its chemical sensing abilities were reported. The inkjet printing-based IDE sensor, fabricated in under 2 min and costing less than USD 0.3, can be adapted as a suitable sensor with rapid and scalable fabrication process capabilities. The effectiveness of the sensor was demonstrated using its ability to detect nitrite in water. The inkjet printing-based IDE sensor effectively quantified 1 ppm of nitrite, which is three times lower than the WHO safe standard, using resistive approach. The fabricated sensor can further be enhanced to sense multiple contaminants simultaneously and be employed for real time water quality assessment. The application of the inkjet printing-based IDE sensor for taste sensing was also investigated. Four out of five taste sensing chemicals were effectively sensed and quantified. The performance of the inkjet printing-based IDE sensor was compared with the DLW-photolithography-based IDE sensor. The DLW-photolithography-based IDE sensor provides IDEs with excellent geometric tolerances and performs better at sensing lower concentrations in taste sensing. However, the inkjet printing-based IDE sensor provides a comparable alternative at a fraction of the cost and time as demonstrated for nitrite sensing. The analysis was carried out at fixed temperatures and environmental conditions; the effect of these external parameters need to be studied in detail.

An important outcome of the reported work is the ability to selectively sense nitrite without the requirement of specific surface modification. This has been demonstrated by real sample analysis with good recovery percentages. The nitrite-sensing platform can be used in real-time applications for the deployment in point-of-source applications. The sensors being developed as a two-electrode setup can be integrated with handheld LCR meters and potentiostats. This concept of point-of-source systems using the developed IDE sensors in conjunction with handheld potentiostats and LCR meters is under development. However, there exist certain limitations which need to be addressed and are currently being worked upon. In its present form, the IDE sensors are capable of sensing individual taste components; however, the sensing of components in a combination of tastes is a shortcoming that needs to be addressed before employing the sensor for real-time applications. Work is being carried out to identify and use surface modification agents for selective detection of taste simulating chemicals. Moreover, a significant limitation identified for these sensors was their inability to sense sodium chloride. Work is being carried out to improve the limiting values of the sensing ranges for the inkjet printing-based IDE sensor and evaluate the sensor for broader application. A further extension of this work could also enable the rapid fabrication of IDEs on substrates suitable for flexible sensing applications.

## Figures and Tables

**Table 1:** Comparative data for nitrite sensing using the developed IDE sensors.

IDE Type	LOD ^1^ (ppm)	LOQ ^2^ (ppm)	Sensitivity (Ohm/ppm)	Sensor Cost ($) ^3^	Fabrication Time (Minutes) ^4^
DLW-Photolithography	0.697	2.322	6.95	1.5	≈40
Inkjet Printed	1.502	5.006	5.54	0.3	≈2

^1^ Limit of Detection; ^2^ Limit of Quantification; ^3^ Component cost based on raw materials, excluding equipment and operation costs; ^4^ Overall process time for fabrication.

## Data Availability

Not applicable.
